# The craziness in losing weight. A systematic review and case report of sibutramine’s psychosis

**DOI:** 10.1192/j.eurpsy.2025.2203

**Published:** 2025-08-26

**Authors:** E. I. Benítez Sánchez, L. Montesinos Almarcha, J. López Álvarez

**Affiliations:** 1 Servicio de Psiquiatría, Complejo Hospitalario Universitario de Badajoz, Badajoz; 2 Servicio de Psiquiatría, Complejo Hospitalario de Mérida, Mérida, Spain

## Abstract

**Introduction:**

Sibutramine is an anorexigenic drug that has been used to treat obesity. After its commercialization, FDA ordered the suspension of its prescription in USA because of the adverse effects. It has structural similarities to amphetamine and is a serotonin-noradrenaline reuptake inhibitor. The primary metabolites are responsible for the activity and inhibit the reuptake of noradrenalin and serotonin from the synaptic cleft. This substance can interfere with one of the known mechanisms of psychosis increasing dopaminergic transmission and leading to potential psychomimetic effects.

**Objectives:**

The main objective in this paper is to obtain a critical evaluation of the literature regarding psychosis and sibutramine. There has been done a bibliographic review in databases such us SCOPUS, Web of Science, UptoDate and PubMed. This review was realized under certain criteria and keywords. The motivations behind this research were generated after an unusual clinical case (in symptomatology’s presentation). Sibutramine is a compound, which is not commercialized in the UE, so the diagnosis of the toxic psychosis was more complicated than the average psychedelics drugs.

**Methods:**

To develop this review, numerous databases were chosen: PubMed, Scopus, UpToDate and Web of Science. However, only in PubMed we have obtain relevant results. For descriptors we have used two terms: “psychosis” and “sibutramine”. In this database, 27 results were obtained. With the criteria previously designed, which were free text access and works published in the last 20 years, only ten of them met all the criteria. With these ten articles, a systematic and qualitative analysis has been done, focusing on the ones which described similar cases.

**Results:**

The sibutramine psychosis is difficult to identify. Frequently is mixed up with other substances in dietary supplements, which can appear as harmless. So, as clinics, we need to be careful, always considering this information in the clinical records. This case was peculiar; a 24 years old woman was consuming sibutramine as a compound in one supplement she was taken for losing weight. Three weeks prior to the consult the symptons started with an erotomaniac delusions, accompanied by disruptive behaviour, mood swings (dysphoria) and irritability. She was hospitalized and treated with antipsychotic medication (aripiprazole) showing a full recovery in five days.

**Conclusions:**

Sibutramine can cause severe mental health disorders. As a compound of dietary supplements, can be easily acquired through online stores. Our work as clinics is to be vigilant of this kind of substances because of the danger that can generate. More studies should be done, to acquire knowledge of the psychobiological mechanisms involved in these cases. The prognosis seems to be positive, but there are certain risks in patients with unknown vulnerability factors.

**Disclosure of Interest:**

None Declared

